# Molecular Insight into the Therapeutic Promise of Flavonoids against Alzheimer’s Disease

**DOI:** 10.3390/molecules25061267

**Published:** 2020-03-11

**Authors:** Md. Sahab Uddin, Md. Tanvir Kabir, Kamal Niaz, Philippe Jeandet, Christophe Clément, Bijo Mathew, Abdur Rauf, Kannan R.R. Rengasamy, Eduardo Sobarzo-Sánchez, Ghulam Md Ashraf, Lotfi Aleya

**Affiliations:** 1 Department of Pharmacy, Southeast University, Dhaka 1213, Bangladesh; 2 Pharmakon Neuroscience Research Network, Dhaka 1207, Bangladesh; 3 Department of Pharmacy, BRAC University, Dhaka 1212, Bangladesh; 4 Department of Pharmacology and Toxicology, Faculty of Bio-Sciences, Cholistan University of Veterinary and Animal Sciences (CUVAS), Bahawalpur 63100, Pakistan; 5 Research Unit, Induced Resistance and Plant Bioprotection, EA 4707, SFR Condorcet FR CNRS 3417, Faculty of Sciences, University of Reims Champagne-Ardenne, PO Box 1039, 51687 Reims CEDEX 2, France; 6 Division of Drug Design and Medicinal Chemistry Research Lab, Department of Pharmaceutical Chemistry, Ahalia School of Pharmacy, Palakkad, Kerala 678557, India; 7 Department of Chemistry, University of Swabi, Anbar 23561, Khyber Pakhtunkhwa, Pakistan; 8 Department of Bioresources and Food Sciences, Konkuk University, Seoul 05029, Korea; 9 Instituto de Investigación e Innovación en Salud, Facultad de Ciencias de la Salud, Universidad Central de Chile, Santiago 8330507, Chile; 10 Department of Organic Chemistry, Faculty of Pharmacy, University of Santiago de Compostela, 15782 Santiago de Compostela, Spain; 11 King Fahd Medical Research Center, King Abdulaziz University, Jeddah 21589, Saudi Arabia; 12 Department of Medical Laboratory Technology, Faculty of Applied Medical Sciences, King Abdulaziz University, Jeddah 21589, Saudi Arabia; 13 Chrono-Environnement Laboratory, UMR CNRS 6249, Bourgogne Franche-Comté University, F-25030 Besançon, France

**Keywords:** Alzheimer’s disease, flavonoids, amyloid plaque, neurofibrillary tangles, PI3K/Akt signaling, MAPK signaling, mTOR

## Abstract

Alzheimer’s disease (AD) is one of the utmost chronic neurodegenerative disorders, which is characterized from a neuropathological point of view by the aggregates of amyloid beta (Aβ) peptides that are deposited as senile plaques and tau proteins which form neurofibrillary tangles (NFTs). Even though advancement has been observed in order to understand AD pathogenesis, currently available therapeutic methods can only deliver modest symptomatic relief. Interestingly, naturally occurring dietary flavonoids have gained substantial attention due to their antioxidative, anti-inflammatory, and anti-amyloidogenic properties as alternative candidates for AD therapy. Experimental proof provides support to the idea that some flavonoids might protect AD by interfering with the production and aggregation of Aβ peptides and/or decreasing the aggregation of tau. Flavonoids have the ability to promote clearance of Aβ peptides and inhibit tau phosphorylation by the mTOR/autophagy signaling pathway. Moreover, due to their cholinesterase inhibitory potential, flavonoids can represent promising symptomatic anti-Alzheimer agents. Several processes have been suggested for the aptitude of flavonoids to slow down the advancement or to avert the onset of Alzheimer’s pathogenesis. To enhance cognitive performance and to prevent the onset and progress of AD, the interaction of flavonoids with various signaling pathways is proposed to exert their therapeutic potential. Therefore, this review elaborates on the probable therapeutic approaches of flavonoids aimed at averting or slowing the progression of the AD pathogenesis.

## 1. Introduction

Alzheimer’s disease (AD) is a form of dementia that most commonly affects older people and is characterized by progressive cognitive decline, which usually starts with a decrease in memory [1,2]. AD is characterized by profound oxidative stress, synaptic connection loss from a specific brain regions, cumulative emergence of intracellular tau pathology, and buildup of extracellular amyloid beta (Aβ) plaques [3,4]. The harmful activities of free radicals and oxidized metabolites in AD include DNA oxidation, lipid peroxidation, and protein oxidation, which ultimately leads to neuronal death [5,6,7,8]. Significant advancement has been achieved to understand AD pathogenesis, since currently available therapeutic methods only deliver moderate relief of cognitive symptoms, such as impairments in perception and memory [9,10,11]. Although, for drug developers, AD has proven to be tremendously challenging, researchers continue to discover better anti-Alzheimer’s treatments. The Food and Drug Administration (FDA) has approved a small number of medications to treat AD; these approved medications are found to improve symptoms, but nonetheless do not change the means of disease advancement and have even exhibited specific undesired effects [12,13]. Biogen Inc. (an American multinational biotechnology company) stopped the phase III clinical trials of the blockbuster anti-Alzheimer’s drug, aducanumab in March 2019, owing to safety concerns [14]. However, in October 2019, they declared that further analysis exposed momentous positive effects for aducanumab in patients with early AD [15]. Owing to the complex nature of AD, there is a growing need for natural substances to treat neurodegenerative events of AD progression [16,17,18,19,20].

In several pathological conditions, for example, neurodegenerative diseases, diabetes, and cancer, natural products endure as a promising source of various molecular characteristics, biochemical specificity, and enormous chemical diversity, which makes these natural products appropriate for the modification of many signaling cascades [21,22,23,24]. Flavonoids are commonly found in various vegetables, fruits, and plants [25,26]. These natural substances are well-recognized as displaying a variety of pharmacological actions [19,27] and also serve as potent metal chelators, free radical scavengers, and antioxidant agents [27,28,29,30]. Flavonoids are also found to subdue the microglial activation, to mediate inflammatory processes in the central nervous system (CNS) [31], to possess potent anti-amyloidogenic, antidepressant effects [32], as well as to improve memory and learning ability [33]. In addition, these natural substances exhibit anti-inflammatory [34,35,36], neuroprotective [37,38], antiaging [39], and anticholinesterase [40] properties.

Acute or chronic administration of flavonoids can penetrate the blood–brain barrier (BBB) signifying that these compounds can operably have a direct impact on the brain. The latter remark suggests that flavonoids can practically have a direct action in the brain; henceforth, these natural substances might be used as a prophylactic, to slow down the advancement of AD [29]. This review thus aims to focus on the molecular mechanisms of plant-derived flavonoids to increase the survivability of neuronal cells in AD and to lower the risk of cellular degeneration. 

## 2. Aging and Age-Associated Changes in the Brain

Out of the pathophysiological alterations that takes place in the aging brain, the major changes that potentially contribute to neurodegeneration include a rise in oxidative stress, loss of neurotrophic support, changes in energy metabolism, changes in protein processing causing a buildup of protein aggregates, dysfunction of the neurovascular system, and activation of the immune system [41,42,43]. Therefore, it is pretty evident that targeting a single alteration will not be effective at averting nerve cell death and damage. Furthermore, there is a strong chance that the extent of the aforesaid alterations will differ among people. Indeed, the latter interact with environmental, lifestyle, and genetic risk factors to varying extents. For example, even though AD is defined in terms of toxic tangle and plaque pathology, it is most commonly related to other harmful processes, for example, inflammation and microvascular damage [44].

Therefore, for the effective prevention of these age-associated alterations of the brain, it is essential to use combination of drugs directed against different targets. Nevertheless, several potential problems are involved with this technique including bioavailability and pharmacokinetic challenges. These problems are even more prominent in case of brain diseases, where it is difficult to get multiple compounds across the BBB and where there are also increased chances of adverse drug–drug interactions. Thus, a better technique would be the identification of small molecules which have several biological actions affecting a multiplicity of age-related pathophysiological alterations, and which play a role in the development and progression of neurodegeneration [45].

## 3. Alzheimer’s Disease Hallmarks

### 3.1. Amyloid Plaques

In the onset and advancement of AD, Aβ plays a crucial role and is considered a vital risk factor [46,47]. Production of the Aβ peptide is typical in healthy people and its production rate (i.e., 7.6%) is normally lower than its rate of clearance (i.e., 8.3%) per hour [48]. However, Aβ might form aggregates under certain conditions and initiate the progression of the disease. In cases of AD and neuronal dysfunctions, there are several shreds of evidence denoting the vital contribution of Aβ [49,50]. An imbalance in Aβ formation and Aβ clearance [51,52] may occur in pathological and aging situations, for example, excitotoxicity and metabolic disorders, which can eventually lead to Aβ accumulation and the formation of senile plaques [53]. In case of AD, the disproportion of the level of Aβ might be because of its disturbance in generation and clearance in the brain. 

In the Alois Alzheimer’s original case report, it was mentioned that both the abnormal extracellular buildup and the deposition of Aβ with 42 or 40 amino acids (i.e., Aβ_1–42_ and Aβ_1–40_) are normal byproducts of the metabolism of the amyloid precursor protein (APP) through an enzymatic sequential cleavage via the β- and γ-secretases in neurons. On the other hand, Aβ42 is more abundant and pathogenic as compared to Aβ40 due to its higher rate of insolubility and fibrillation within the plaques [54].

### 3.2. Neurofibrillary Tangles

Tau is an abundantly found neuronal microtubule-associated protein which is produced by neurons. Tau is localized in the axons and the cell body of neurons [55]. During neuronal development, the expression of tau is increased by the nerve growth factor under normal circumstances [56]. However, tau is also generated via glial cells under various pathological conditions [57,58]. CNS is the prime region of tau expression, while its mRNA can also be found in peripheral tissues [58]. It is assumed that neuronal death and production of NFTs are the results of tau abnormalities, which can ultimately cause dementia [59]. In the somatodendritic region of the neurons, tau accumulates in its hyperphosphorylated forms [56]. On the other hand, NFTs formation is directly linked with neuronal dysfunction; furthermore, the number of NFTs is associated with the extent of dementia in AD [60].

In the Alois Alzheimer’s original autopsy case report, it was first stated that within the perikaryal region of pyramidal neurons, NFTs were mentioned as intraneuronal filamentous inclusions. It was revealed through ultrastructure experiments on AD brain specimens that NFTs are primarily composed of paired helical filaments (PHFs), which are fibrils of approximately 10 nm in diameter that form pairs with a helical three-dimensional conformation and a regularly repeated pattern of almost 65 nm [61,62,63]. Interestingly, the presence of fibrils inside the NFTs in a small proportion does not cause the formation of pairs but leads to straight filaments without the repeated pattern of PHFs [64]. A study reported the existence of twisted ribbon-like assemblies of tau fibrils in in vitro models, therefore challenging the theory of PHFs [65].

## 4. Proteolytic Processing of APP and Aβ Production

**Figure 1:**
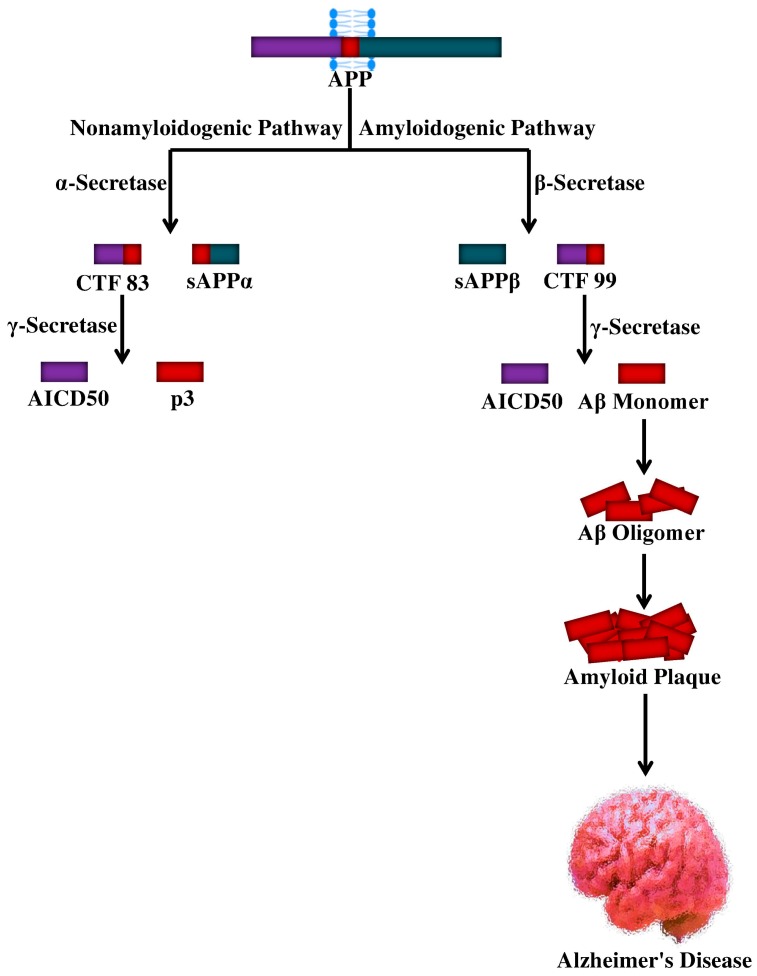
Cellular processing of amyloid precursor protein and the genesis of Aβ peptide. APP, amyloid precursor protein; sAPP, soluble amyloid precursor protein; CTF, C-terminal fragment; Aβ, amyloid beta; AICD, APP intracellular domain; p3, peptide p3.

The APP is a type I transmembrane protein containing a large extracellular domain. Furthermore, it includes a hydrophobic transmembrane domain and a short C-terminus, designated the APP intracellular domain (AICD), which can go through alternative splicing and can give rise to at least eight isoforms of APP [66]. APP can be proteolytically processed via two distinct pathways such as the amyloidogenic pathway and the non-amyloidogenic pathway with the help of the α-, β-, and γ-secretases [67], as shown in Figure 1. The biochemical characteristics of these secretases have been elucidated. It has been proposed that three members of the disintegrin and metalloprotease (ADAM) family; the metalloproteinases ADAM10, ADAM9, and the ADAM17/tumor necrosis factor-α converting enzyme (TACE) [68,69] may exhibit α-secretase activity. On the other hand, the β-secretase activity has been mainly ascribed to the β-site APP-cleaving enzyme (BACE1) [70,71,72,73]. Subsequent cleavage by the α-secretase and γ-secretase complex prevents the formation of Aβ in the non-amyloidogenic pathway. In fact, α-secretase cleavage originates the membrane-linked C-terminal fragment consisting of 83 amino acids (CTF83), which is subsequently cleaved via the γ-secretase complex, ultimately leading to AICD and P3 peptide [74]. 

Interestingly, as per the amyloidogenic pathway, the β-secretase can process the APP to generate a membrane-linked C-terminal fragment consisting of 99 amino acids (CTF99) and a soluble APPβ (sAPPβ) fragment. The former sAPPβ is also a substrate for the γ-secretase complex, the cleavage of which leads to AICD release and Aβ generation, which can span from 1–38 to 1–43 residues. Though Aβ_1–42_ is a minor species, Aβ_1–40_ is mainly generated under non-pathological circumstances [75,76]. APP trafficking and processing can be influenced by various factors, including Aβ itself [77,78,79] and stress conditions [80,81]. Along with the formation of NFTs and the hyperphosphorylation of tau [82], several pathological events including neuroinflammation, oxidative stress, apoptosis, and neurotoxicity can take place due to excessive production of Aβ [83,84,85]. In certain brain areas of AD individuals, the abnormalities mentioned above can lead to neuronal loss and synaptic damage, further causing the progression of the disease [86].

## 5. Phosphatases and Kinases for Tau Phosphorylation

**Figure 2:**
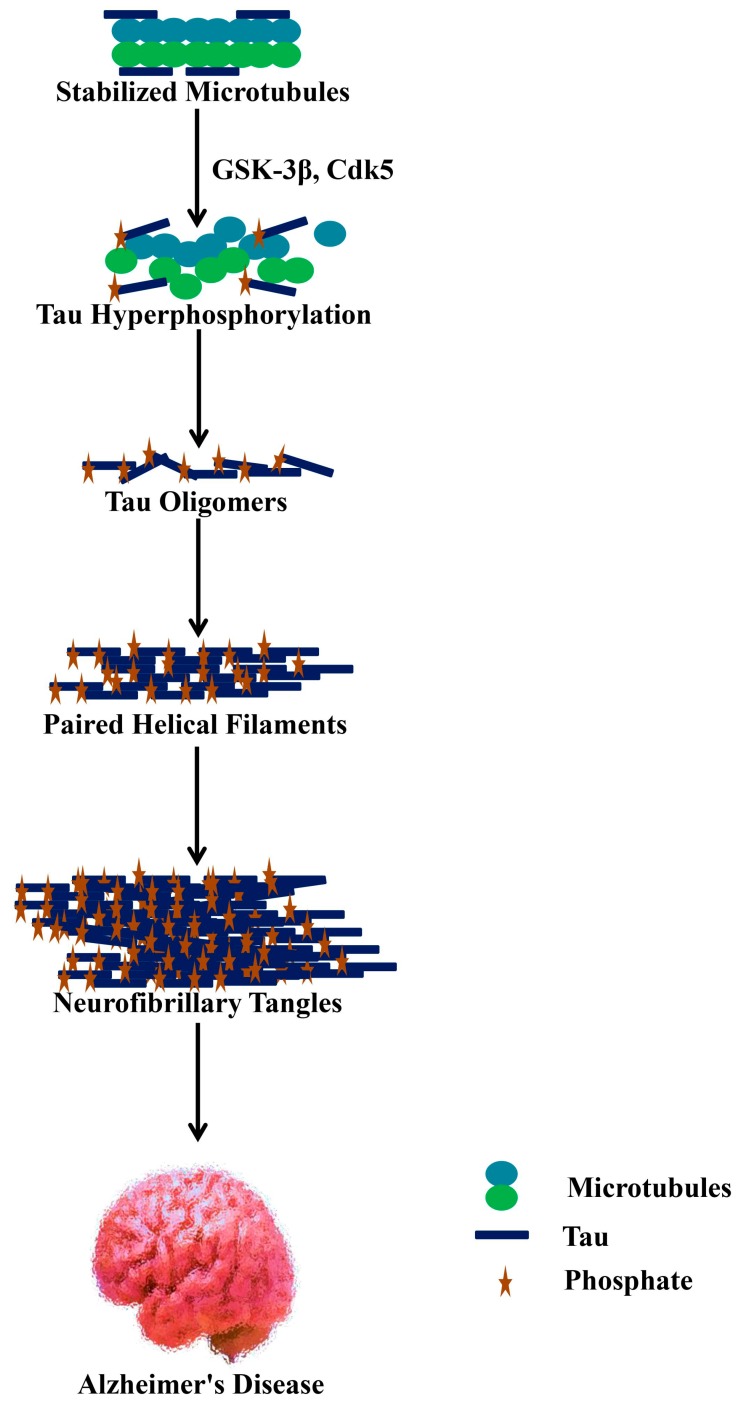
Hyperphosphorylated tau dissociates from microtubules and depolymerizes as well as aggregates as neurofibrillary tangles. GSK-3β, glycogen synthase kinase 3β; Cdk5, cyclin-dependent kinase 5.

As was previously evocated, the hyperphosphorylation of tau is another hallmark of AD. Tau phosphorylation controls its binding action to microtubules triggering their assembly. On the other hand, tau loses its biological activity in the hyperphosphorylated state [87], basal levels of phosphorylation being essential for optimum tau effect (Figure 2). It has been reported that twenty-eight phosphorylation sites of tau (i.e., among approximately eighty-five phosphorylation sites) are mainly phosphorylated in AD brains [88].

It is supposed that the downregulation of tau phosphatase(s) or the upregulation of tau kinase(s) in case of AD can cause abnormal phosphorylation of tau, even though both events might not be mutually exclusive [58]. cAMP-dependent protein kinase (PKA), calcium/calmodulin-dependent kinase II (CaMK-II), cyclin-dependent kinase 5 (Cdk5) and glycogen synthase kinase 3β (GSK-3β) are the kinases that are predicted to have a substantial contribution to the phosphorylation of brain tau [89]. Interestingly, among tau kinases, GSK-3β might have a significant impact on controlling the phosphorylation of tau under both pathological and physiological conditions. Furthermore, GSK-3β can cause the phosphorylation of tau at multiple residue levels. Protein phosphatases (PP) 1, PP2A, PP2B (calcineurin), and PP2C are all potential candidates that can dephosphorylate tau [90]. As a whole, when compared to non-demented older adults, tau is at least 3- to 4-times more hyperphosphorylated in the brain of AD patients [91].

## 6. Flavonoids

**Figure 3:**
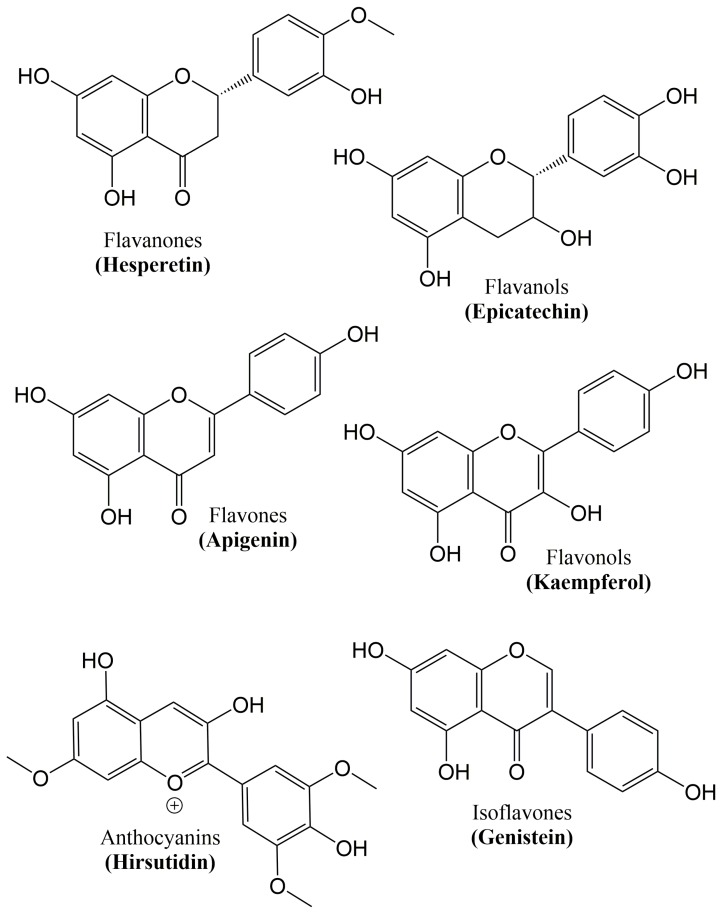
Chemical structure of the major classes of flavonoids.

Growing evidence confirms that flavonoids display promising neuroprotective potential due to their ability to lessen the progression of age-related neurodegenerative disorders or avert the onset of neurodegeneration [92,93]. Their attitudes to influence learning and cognition in animal models of disease and also in humans have been exhibited by dietary supplementation experiments involving flavonoid-rich food or plant extracts [94,95,96,97,98]. In the brain, the possible beneficial effect of flavonoids appears to be linked to their potential to interact with glial signaling and intracellular neuronal pathways, therefore triggering neuronal regeneration, increasing existing functions of the neurons, protecting vulnerable neurons, or affecting the cerebrovascular and peripheral system [99]. In plants, flavonoids are naturally-occurring polyphenolic compounds [92,100]. Furthermore, they can be found in beverages and foods of the plant source, for example, a range of vegetables, fruits, wine, tea, cereals and cocoa [101,102]. The six major subclasses of flavonoids [103] are given in Table 1 and Figure 3.

It was assumed that the potential role of flavonoids in stimulating cognitive functions, learning, and memory is mediated by their antioxidant capabilities [104]. However, growing evidence shows that these natural substances are able to interact with the molecular and cellular components of the brain accountable for memory. Flavonoids have the potential to encourage neurogenesis, trigger neuronal regeneration, increase existing neuronal function, and prevent neuronal dysfunction (Table 2) [104,105].

## 7. Role of Flavonoids on Alzheimer’s Hallmarks

### 7.1. Aβ Neuropathology

**Figure 4:**
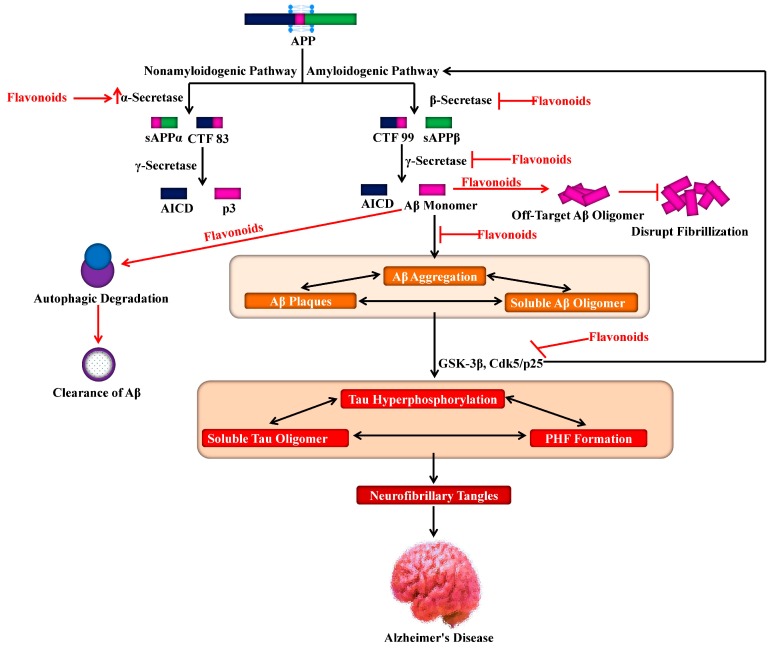
Role of flavonoids in targeting amyloid precursor protein processing for lessening Alzheimer’s pathogenesis. APP, amyloid precursor protein; sAPP, soluble amyloid precursor protein; CTF, C-terminal fragment; Aβ, amyloid beta; AICD, APP intracellular domain; p3, a peptide; GSK-3β, glycogen synthase kinase 3β; Cdk5, cyclin-dependent kinase 5.

A variety of flavonoids have been reported to reverse cognitive impairments and to inhibit AD development, suggesting their potential for therapeutic applications [114,115,116]. Various experiments have focused on the anti-amyloidogenic properties of flavonoids (Figure 4), as a critical natural remedy to lessen AD [117,118]. Henceforth, it has recently been proven in APP/PS1 mouse models of AD that blackcurrant and anthocyanin-enriched bilberry extracts can reduce behavioral abnormalities associated with AD and regulate APP processing [119]. On the other hand, transgene-linked defective spatial reference memory and behavioral impairment in a transgenic PSAPP mouse model of cerebral amyloidosis were averted with orally administered tannic acid for six months. Additionally, several different experiments have reported on the efficacy of flavonoids in learning and memory. A citrus flavonoid, nobiletin, was found to reduce the burden of Aβ and plaques in the hippocampus region, thus improving the memory deficits induced by Aβ in a transgenic AD mouse model [120]. It was also reported that reduced cognitive impairment coincides with decreased levels of high-molecular-weight soluble Aβ oligomers upon oral administration of grape-derived polyphenols for five months in the brain of Tg2576 mice [121].

The citrus flavonoid, luteolin was found to reduce the processing of APP by the amyloidogenic γ-secretase activity and decrease the generation of Aβ in both human Swedish mutant APP transgene-bearing neuron-like cells and primary neurons [122] as shown in Figure 4. Furthermore, the deposition of Aβ was averted due to the administration of curcumin or polyphenol-rich grape seed extracts for nine months in the brain of AD mouse models [123].

In a transgenic AD mouse model, it was found that long-term administration (i.e., sixteen months) of *Ginkgo biloba* extracts considerably decreased the levels of APP, further proposing the potential neuroprotective properties of these extracts associated to APP-reducing activities [124]. It has also been reported that cerebral vascular and brain parenchymal Aβ deposits were reduced in tannic acid-treated PSAPP mice, signifying that tannic acids play a role as natural inhibitors of β-secretase [125]. On the other hand, the reduction in secreted Aβ levels and active inhibition of BACE-1 activity were observed in primary cortical neurons following the use of natural flavonoids [126]. Epigallocatechin-3-gallate (ECG) and curcumin were found to reduce Aβ-mediated BACE-1 upregulation in neuronal cultures [127].

Several experiments have been directed toward identifying the beneficial properties of regular green tea intake. It has indeed been demonstrated that a green tea polyphenol such as ECG has a beneficial contribution in terms of reducing brain Aβ levels through the control of the APP processing [128,129]. Interestingly, ECG causes elevation of the nonamyloidogenic processing of APP by enhancing α-secretase cleavage [130]. It was also reported that ECG arbitrated the augmentation of the non-amyloidogenic APP processing via ADAM10 maturation through an estrogen receptor-α/phosphoinositide 3-kinase/Ak-transforming-dependent mechanism. Modulating selective estrogen receptors might be a therapeutic target, as a decrease in the level of estrogens after menopause is associated with an elevated risk of AD development [131]. On the other hand, ECG might be considered in the treatment and prophylaxis of AD as a substitute for estrogen therapy [132].

Since ECG possesses the ability to reduce the formation of the β-sheet-rich amyloid fibrils, it might have a neuroprotective effect. It has been confirmed that this compound reduces the Aβ fibrillogenesis via its direct binding to the natively unfolded polypeptides thus averting their conversion into toxic intermediates [133]. Interestingly, it has been observed that ECG has the power to convert large Aβ fibrils into smaller ones, amorphous protein aggregates that are non-toxic in nature. This phenomenon signifies that ECG is a powerful remodeling agent for amyloid fibrils [134]. Additionally, other flavonoids also exhibited anti-amyloidogenic features, particularly myricetin, which displayed anti-amyloidogenic activity in in vitro models via reversibly and specifically binding to the amyloid fibril structure of Aβ, instead of monomers of Aβ [135,136]. In general, these experiments report that specific flavonoids can disturb fibrillation by leading to the generation of off-target Aβ oligomers (Figure 4), and function by increasing the activity of ADAM10, or act as BACE-1 inhibitors, subsequently decreasing the production of Aβ.

Most of the consumed dietary polyphenols do not get absorbed by the upper intestinal tract. Gut microbiota helps in breaking these dietary polyphenols into low-molecular-weight phenolic compounds in the colon, which are more effectively absorbed by the gastrointestinal epithelial cells [137,138]. A study has revealed that the administration of grape seed polyphenol extracts in mice caused the formation of 11 unique polyphenol metabolites as measured in urine, four metabolites in the plasma, whereas only two metabolites, 3-(3′-hydroxyphenyl) propionic acid and 3-hydroxybenzoic acid, were detected in the brain following perfusion [139]. Both 3-(3′-hydroxyphenyl) propionic acid and 3-hydroxybenzoic acid are likely derivatives of the flavonol quercetin, and are generated following ring cleavage of the latter by *Enterobacter* spp. in the gut and enterocyte phase II modification, for instance, dehydration or reduction [140]. In the study of Wang et al. [141], it was reported that 3-(3′-hydroxyphenyl) propionic acid and 3-hydroxybenzoic acid have a strong ability to attenuate Aβ oligomerization in AD.

Nevertheless, further experiments are needed to identify which flavonoid structures contain potent beneficial properties and their underlying mechanisms of action. In a recent review, three structural characteristics of natural products have been proposed to explain their inhibitory activity against the aggregation of Aβ42 [142]. The first characteristic is that carboxy acid derivatives with anthraquinoids or triterpenoids which are able to produce a salt bridge with basic amino acid residues such as Lys28 and Lys16 in the Aβ42 trimers or dimers. The second characteristic involves non-catechol-type flavonoids with molecular planarity because of α,β-unsaturated carbonyl groups that have the ability to interact with the intermolecular β-sheet region in Aβ42 aggregates, particularly aromatic rings, such as those of Phe20 and Phe19. The third characteristic includes catechol-type flavonoids that can produce Michael adducts with the side chains of Lys28 and Lys16 in monomeric Aβ42 by flavonoid autoxidation [142].

### 7.2. Tau Neuropathology

In AD, flavonoids might contribute to downstream targets, for instance, phosphorylation of tau. Relating to this, several experiments have explained the characteristics of the action of flavonoids in tau, which might influence AD. In the transgenic AD mouse model, the administration of ECG was found to modulate the profiles of tau, along with prominent suppression of the phosphorylated tau isoforms (i.e., sarkosyl-soluble) [129]. In contrast, epicatechin-5-gallate and myricetin were found to inhibit the heparin-mediated formation of tau [143]. Other experiments using grape-derived polyphenols have revealed their aptitude to hinder the neuropathology of tau in an AD mouse model, delaying aggregations of tau, and disrupting PHFs and dissociating preformed tau aggregates [142,143,144,145,146,147].

Various kinases including GSK-3β, play a role in tau phosphorylation and are involved in AD pathogenesis. It has been observed that the activities of several kinases are suppressed by flavonoids, and therefore the latter can help in AD prevention. In this regard, for example, activities of protein kinases including GSK-3β and Cdk5/p25 can be inhibited by indirubins, which are byproducts of bacterial metabolism. These two protein kinases (Figure 4) are involved in abnormal tau phosphorylation, which is observed in AD patients [148]. It has been reported that morin, a flavonoid, can inhibit GSK-3β-mediated tau phosphorylation and can also suppress the activity of GSK-3β. Additionally, morin gives protection against Aβ-induced neurotoxicity in human neuroblastoma cells and also reduces the tau phosphorylation mediated by Aβ. Moreover, in the hippocampal neurons of transgenic animals (3xTg-AD mice), treatment with morin was shown to cause a reduction in tau hyperphosphorylation [149]. It has also been observed that cyanidin-3-*O*-glucoside provided marked protection against cognitive dysfunctions stimulated by Aβ administration in animal models, which is mediated by modulation of GSK-3β/tau [150]. On the other hand, luteolin is found to cause a reduction in GSK-3 activity and in the levels of soluble Aβ as well as disruption in PS1-APP association [122].

## 8. Role of Flavonoids in the Signaling Pathways of Alzheimer’s Disease

It has been indicated through copious evidence that certain flavonoids [151] and their various metabolites [152,153,154] can exert useful actions on neurological processes via their interaction with various neuronal signaling pathways. The tyrosine kinase receptor B (TrkB) [155], as well as nicotinic acetylcholine [156,157], δ-opioid [158,159], type A gamma-aminobutyric acid (GABA_A_) [160,161,162], adenosine [163], testosterone [164] and estrogen [132] receptors, represent potential flavonoid-binding sites on neurons [165]. Interestingly, flavonoids and their metabolites were found to exert effects on neurons via their interactions with various protein kinase- and lipid kinase-signaling cascades, for instance, the nuclear factor kappa-light-chain-enhancer of activated B cells (NF-κB) pathway as well as the protein kinase C (PKC), tyrosine kinase, phosphoinositide-3-kinase (PI3K)/Akt, and mitogen-activated kinase (MAPK) signaling pathways [104,105,152,166,167,168,169,170,171,172].

MAPKs are found to control several cellular processes by transducing extracellular signals into intracellular responses [173,174]. On the other hand, flavonoids and their metabolites can selectively interact with the MAPK signaling pathways [169,175] through their interaction with some MAPK kinases such as MAP kinase 1 (MEK1) as well as MEK2 and membrane receptors [169,176,177]. The effect of flavonoids on the extracellular signal-regulated kinase (ERK) pathway seems to be facilitated by these kinases [152,171,178,179,180,181]. Flavonoids contain a close structural homology to some specific pharmacological modulators of the ERK signaling pathway, for instance, PD98059 (2′-amino-3′-methoxyflavone, (MEK1 inhibitor)). It has also been observed that activation of the cAMP response element-binding protein (CREB) can take place due to ERK activation; eventually, this can cause an upregulation in neuroprotective pathways and alterations in memory and synaptic plasticity [182,183]. Blueberry contains high amounts of anthocyanins and flavanols. A study has revealed that the memory performance of rats supplemented with blueberry was associated with CREB activation and with rises in both mature and pro levels of the brain-derived neurotrophic factor (BDNF) in the hippocampus [184], both of which were found to be linked to the regulation of long-term memory and synaptic plasticity. Furthermore, in senescence-accelerated mouse prone-8 mice, the intake of green tea catechins for six months averted memory deficits and spatial learning by enhancing the action of the protein kinase A/CREB pathway, reducing Aβ_42_ oligomers, and through the upregulation of synaptic plasticity-related proteins in the hippocampus [185]. Likewise, flavonoids can cause stabilization of the nuclear factor erythroid 2-related factor 2 (Nrf2) and the hypoxia-inducible factor-1 [186], which act as modulators of PPAR-γ [187] and activate the PGC-1α [188] pathway. An alteration in these molecular pathways by flavonoids attenuates AD progression with a lessening of the oxidative stress, the improvement in mitochondrial dysfunction, the reduction in insulin resistance and an amelioration of the memory injury.

**Figure 5:**
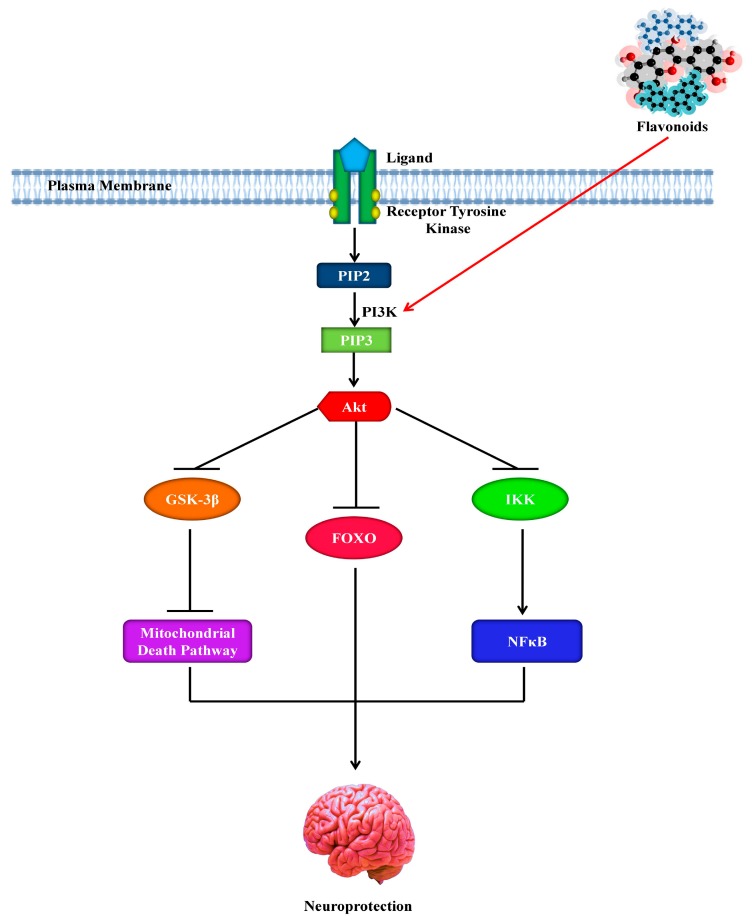
Neuroprotective effect of flavonoids by activating the phosphoinositide 3-kinase pathway. PI3K, phosphoinositide 3-kinase; GSK-3β, glycogen synthase kinase 3β; Akt, protein kinase B; PIP2, phosphatidylinositol-3,4-biphosphate; PIP3, phosphatidylinositol-3,4,5-triphosphate; FOXO, forkhead box; NFκB, nuclear factor κB; IKK, IκB kinase.

In addition, flavonoids regulate PI3K through direct interactions with their adenosine triphosphate (ATP)-binding site [167,189] as shown in Figure 5. Based on the structure of quercetin, the most selective PI3K inhibitor, LY294002, was modeled [170,190]. Quercetin and LY294002 [191] fit into the binding pocket of the enzyme, although in various directions [191]. The extent of unsaturation of the C2–C3 bond in the C- ring and the number of hydroxyl (-OH) group substitutions on the flavonoid B-ring are crucial determining factors of this particular bioactivity. Furthermore, it seems that cellular responses can vary from one flavonoid to another, reliant on their degree of interaction with either downstream kinases or receptors, suggesting possible structure-dependent signaling pathways.

Regarding this, the flavanone, hesperetin, for instance, has been found to cause activation of the Akt/protein kinase B (PKB)-signaling pathway to confer prosurvival properties in cortical neurons [181], while the flavonol, quercetin and its specific in vivo metabolites were found to modulate the prosurvival Akt/PKB- and ERK1/2-signaling pathways by hindering the activity of PI3K [152]. ECG has been found to stimulate ERK- and PI3K-dependent elevation in the phosphorylation of CREB and to upregulate the levels of the ionotropic glutamate receptor 2 (GluR2) in cortical neurons and can therefore play a vital role as a modulator in synaptogenesis, plasticity, and neurotransmission [192]. It has been reported that adding blueberry to the diet of aged animals for twelve weeks can cause an increase in expression of the activity-regulated cytoskeletal-associated protein (Arc/Arg3.1), induction of hippocampal Akt phosphorylation, and activation of the downstream mammalian target of rapamycin (mTOR) [184]. As it has been suggested that Arc, under the regulatory control of both BDNF, is essential in long-term potentiation (LTP) [193], such alterations might underlie events associated to the spatial memory via the induction of morphological modifications and the facilitation of alterations in synaptic strength [194]. Interestingly, modifications in neuronal spine density and morphology are regarded as necessary for memory and learning [195]. Some studies have indicated that changes in neuronal morphology can take place due to supplementation with flavonoids [180,196] and that the neuronal dendrite outgrowth in vitro can be affected by certain flavonoids [197].

NFTs accumulation and the hyperphosphorylation of tau are found to be strongly associated with cognitive impairments. In the case of AD pathogenesis, among the kinases that can phosphorylate tau, GSK-3β is intensely considered [148,198]. Indeed, it has been demonstrated that flavonoids exhibit beneficial effects through their inhibition of the action of certain kinases that play a role in AD pathology, as previously stated. Therefore, in the case of AD, it appears to be sensible to conclude that disproportions in the phosphorylation system are thus one of the reasons for cytoskeletal protein hyperphosphorylation. Although there is no proof of the fact that flavonoids might influence signaling pathways through regulation of the activity of phosphatases, strong evidence is given for the capability of flavonoids to regulate kinases. It is possible that alterations in the activation of ERK and associated transcription factors might result from the flavonoid-mediated regulation of the activity of phosphatases, as phosphatases are integral to many signaling pathways and reverse the effects of kinases [151]. However, further studies are required to appraise the flavonoid’s potential to activate or inhibit phosphatases and their mechanisms of action.

## 9. Role of Flavonoids in Autophagy

The human body uses autophagy in order to clean out damaged cells to regenerate healthier and newer cells [199,200]. Furthermore, autophagy also controls the production and the clearance of Aβ [201]. To reduce synaptic defect and neuronal death, clearance of Aβ from the brain is another main target for anti-AD drugs [202]. In the study of Pierzynowska et al. [203], it was shown that at a high dose (i.e., 150 mg/kg/day), genistein caused activation of autophagy in a streptozotocin-induced rat model of the sporadic AD. Moreover, at this high dose, the authors also noticed that genistein triggered the complete degradation of Aβ and hyperphosphorylated tau via induction of autophagy [203].

The flavonoid, wogonin has also been reported to display the ability to promote the clearance of Aβ (Figure 4) in the primary cortical astrocytes and attenuate secretion of Aβ in the SH-SY5Y cells that overexpress *BACE1* and *APP*, via the mTOR/autophagy signaling pathway [204]. On the other hand, wogonin has the ability to suppress the P-glycoprotein, which is an ATP-binding cassette export protein with the function of transporting the drugs back into the blood. Nevertheless, the ability of this compound to penetrate the BBB is not confirmed by any direct evidence [205]. Interestingly, hesperetin and its glycoside hesperidin are other flavonoid compounds that can provide protection, through downregulation of the Aβ-activated autophagy, to neuronal cells against Aβ-stimulated impairment of glucose transport and glucose uptake [206]. Importantly, it has been reported in an in vitro ECV304/C6 monolayer co-culture BBB model, that hesperetin has the ability to penetrate the BBB [207]. Interestingly, quercetin can reduce Aβ aggregation and the related paralysis via proteasomal degradation pathways and the activation of macroautophagy [98].

Chronic unpredictable mild stress is a causal factor in cases of neurodegenerative diseases, especially in AD, since it can lead to the accumulation of Aβ and can also impede hippocampus-related memory and learning processes [208]. Interestingly, chronic unpredictable mild stress can be alleviated by ECG [208]. It has been observed that the activity of ECG is facilitated by the restoration of the autophagy flux in these brain areas, which further avoids those symptoms and impedes the progression of chronic unpredictable mild stress [208].

Silibinin is a flavonoid isolated from *Silybum marianum*. Silibinin improves the depressive behaviors induced by Aβ42 in rats and also alleviates neuronal damage by suppressing autophagy in the hippocampus [209]. Furthermore, this compound has also been found to protect neurons via suppressing both autophagic cell death and the mitochondrial pathways [210]. It has been reported by Jeong et al. [211] that naringin weakens autophagic stress in the kainic acid-treated hippocampus in vivo, which was verified by the expression of the microtubule-associated protein light chain 3.

## 10. Role of Flavonoids in Neuropathological Insults

Due to metabolic events, several inappropriate events such as neuroinflammation, depletion of endogenous antioxidants, glutamatergic excitotoxicity and neurotoxicity occur as neurodegenerative outcomes in AD [212]. Various studies have revealed that the use of flavonoids helps to attenuate neuronal injuries and lessen the progression of neurodegeneration through regulating the components of kinase-signaling cascades, for example, the PI3K/Akt, PKC, and MAPK pathways [213,214]. ECG attenuates substantia nigra pars compacta damages through free radical chelation as well as regulating various signaling pathways such as PKC and PI3K which are responsible for neuroprotection [215,216].

## 11. Role of Flavonoids as Free Radicals’ Scavengers

During metabolic processes, various types of free radicals are produced. When free radicals are produced in excess, normal physiological processes are disturbed which leads to lipid peroxidation, protein degradation, DNA damage and deregulation of different chemokines [217]. Besides, these free radicals are also involved in the neuroinflammatory damage which proceeds to AD development [218,219]. A high level of oxidative stress biomarkers gives an indication of AD progression [220]. Furthermore, various studies established that patients of AD display low antioxidant power in their plasma [221,222]. Moreover, in the transgenic animal model of AD, high lipid peroxidation and protein degradation were reported [223]. Microglia activation is not only responsible for the production of pro-inflammatory cytokines, it also enhances the superoxide anion levels via the NADPH oxidase (NOX) which gives clues toward neurodegenerative events and AD [224,225,226].

Green tea contains strong antioxidants such as catechins and related polyphenols; these phytochemicals function as chelators of metal ions and scavengers of free radicals. ECG is responsible for the donation of electrons to the ROS-induced free radicals, thus inhibiting DNA damages linked to oxidative stress [227]. During lipid peroxidation by the iron ascorbate (i.e., labile ferri ferro complex), green tea lessens the progression of chain reactions in the mitochondrial membrane of brain cells. Due to the capacity of epigallocatechin gallate (EGCG) to inhibit fibril formation during Aβ aggregation, it is considered as an effective scavenger among the catechins [228]. EGCG helps to attenuate lipid peroxidation that occurred due to Aβ [229,230]. It also lessens Aβ-induced programmed cell death and caspase activity, which enhances the survival of hippocampus neurons [229].

Quercetin is a strong antioxidant which has the ability to decrease the levels of superoxide anion free radicals. Thus, quercetin is useful in the management of multiple diseases, including AD [231]. In the study of Zhu et al. [232], 14 flavonoids were isolated and identified from *Agrimonia pilosa* Ledeb. Among these flavonoids, seven display significant 2,2-diphenyl-1-picrylhydrazyl (DPPH) free radical scavenging activities, namely luteolin-7-*O*-β-glucoside, rutin, hyperoside (3-*O*-quercetin galactoside), quercitrin, quercetin, luteolin and catechin with IC_50_ values of 8.12, 6.36, 6.34, 7.12, 4.36, 7.29 and 5.06 µM, respectively [232]. It has been revealed using the DNA nicking assay, that five flavonoids (i.e., rutin, quercitrin, hyperoside, catechin, and taxifolin) exhibited a significant protective effect against oxidative deoxyribonucleic acid damages [232]. A DPPH free radical scavenging activity has also been observed with the methanolic extracts from the stem bark of *Artocarpus gomezianus* [233]. Four flavonoids were isolated using the bio-assay guided fractionation of those methanolic extracts. These four flavonoids, including (+)-catechin, artobiloxanthone, cycloartobiloxanthone, and artonin E, exhibited significant DPPH free radical scavenging activity as well as showing a significant inhibitory activity on the production of nitric oxide in murine macrophage-like cells [233].

## 12. Role of Flavonoids as Cholinesterase Inhibitors

Acetylcholine (ACh) is the neurotransmitter responsible for the signaling among synapses, which is degraded via enzymes such as acetylcholinesterase (AChE) and butyrylcholinesterase (BChE) [234]. As there are copious studies reporting low levels of ACh in AD brains [47,235,236], cholinesterase inhibitors thus represent the best therapeutic remedy to increase ACh levels at synaptic junctions [237]. Various studies have reported the use of flavonoids such as kaempferol, genistein, apigenin, quercetin, naringin, diosmin, silibinin, silymarin, as possible inhibitors of AChE and BChE. Among all these compounds, quercetin was found to exert the highest activity (i.e., 76.2% of AChE inhibition), which is significantly higher compared to silibinin, genistein and luteolin (i.e., 51.4% and 65.7, and 54.9% of BChE inhibition, respectively) [238]. The study of Uriarte-Pueyo and Calvo [239] systematically summarized 128 flavonoids displaying an AChE-inhibiting activity, making flavonoids symptomatic anti-Alzheimer agents.

A research group has isolated and screened several potent flavonoids with cholinesterase inhibitors activity from the aerial part of *Achillea millefolium* (yarrow) [240]. It was shown that 6-OH-luteolin 7-*O*-β-d-glucoside had significant in silico and in vitro BChE and AChE inhibitory effects with an IC_50_ of 1.97 and 1.65 μM, respectively, in comparison with a standard of neostigmine (IC_50_ 4.36 and 1.08 μM). Collectively, these findings suggest that 6-OH-luteolin 7-*O*-β-d-glucoside might be developed as a novel therapeutic agent for AD management [240]. Another group has isolated 13 flavonoid derivatives along with two ginkgolides from the leaves of *Ginkgo biloba*. These compounds were screened to assess their possible acetylcholinesterase inhibitors (AChEIs) activity in vitro. It was quite evident from their IC_50_ values (ranging from 57.8 to 133.1 μg/mL) that all these 13 derivatives showed significant AChEI activity in a dose-dependent manner as compared to the standard chlorpyrifos (i.e., an organophosphate insecticide, IC_50_ 12.4 μg/mL). However, it was observed that the two ginkgolides (i.e., the ginkgolide B and C) were inactive against AChE [241]. Another study also reported the screening of several flavanols, flavanones, flavonols, isoflavones, and flavones for their inhibitory effect against *Electrophorus electricus* AChE [242]. Among all the studied compounds, the flavone baicalein exhibited the highest efficacy as an AChEI. The IC_50_ of this flavone was 0.61 μM in comparison with the standard tacrine (IC_50_ 25.4 μM) [242]. 

## 13. Role of Flavonoids as Cognition Enhancers

Flavonoid-rich food items have enormous biological effects on memory [104,243,244]. The isoflavones derived from soy as well as soy-derived foods are effective in learning and perception via mimicking the estrogen activity in the brain [245]. Furthermore, isoflavones also control the concentrations of ACh as well as various neurotrophic factors such as the nerve growth factor and the BDNF in the frontal cortex, as well as the hippocampus of the brain involved in cognitive function [246,247].

Grape juice, cocoa, and blueberry are used as memory-enhancers due to the presence of flavonoids [248,249,250]. Various studies have suggested that the administration of pure quercetin and EGCG, along with daily usage of flavonoid-rich fruits such as blueberry, pomegranate, strawberry, and grapes, enhance cognition, also affecting memory acquisition, retention, and recovery as well as short and long term memory [251,252]. However, there are fewer studies on cognition and spatial working memory in animal models fed with fruits with high flavonol and anthocyanin contents [253,254]. Furthermore, it has been noted that EGCG enhances the progression of the spatial memory [180], while the flavonoids from blueberry display the same action via their action on the dentate gyrus, which are most sensitive to the effects of aging [255,256]. Blueberry flavonoids lead to an enhancement of precursor cell proliferation in the dentate gyrus of animal models, which ultimately improves dentate gyrus neurogenesis and cognition [257].

Ettcheto et al. [258] have assessed the possible beneficial activity of EGCG in a well-established preclinical mixed model of familial AD and type 2 diabetes mellitus, based on the use of transgenic APPswe/PS1dE9 mice fed with a high-fat diet. Interestingly, treatment with EGCG improved memory impairments and insulin sensitivity. Additionally, EGCG significantly elevated the synaptic markers and rates of cAMP response element-binding phosphorylation by a reduction in the activation of the unfolded protein response (UPR), through the decrease in the posterior downregulation of protein tyrosine phosphatase 1B (PTP1B) as well as in the levels of the activation factor 4. EGCG also markedly reduced Aβ generation in the brain and decreased the plaque burden by elevating the α-secretase levels [258]. Unfortunately, the inherent instability of EGCG limits its effectiveness and bioavailability. Cano et al. [109] stated that oral treatment with dual-drug-loaded PEGylated PLGA (EGCG/ascorbic acid) caused the accumulation of EGCG in all major organs, including the brain, in a mouse model study. Interestingly, in APPswe/PS1dE9 mice, the oral administration of EGCG/ascorbic acid nanoparticles caused a significant rise in synapses, as evaluated by the expression of synaptophysin, a decrease in neuroinflammation and Aβ plaque burden, as well as a reduction in the cortical levels of insoluble and soluble Aβ42. Moreover, these morphological alterations took place alongside marked enhancement in spatial memory and learning [109].

## 14. Conclusions

In natural foods, flavonoids are broadly available, and therefore AD treatments with such natural compounds via dietary supplements or diet can be regarded as an attractive substitute. In a range of animal and cell culture models, flavonoids have confirmed their beneficial effects against the AD pathogenesis. However, before putting novel flavonoid-based dietary applications in practice to lower the risk of AD, further studies are required which will address the specific processes through which flavonoids exhibit their potential neuroprotective effects. In order to develop novel approaches for neuroprotection, understanding the processes underlying flavonoid–protein interactions in AD may represent an auspicious objective.

## Figures and Tables

**Table 1:** Classification of flavonoids and their dietary sources.

Class	Flavonoids	Dietary Sources
Flavanones	Naringin, Naringenin, Hesperetin, Eriodictyol	Tomatoes, grapefruits and citrus fruits
Flavanols	Epigallocatechin gallate, Epigallocatechin, Epicatechin, Catechin	Cocoa, red wine, grapes, and green tea
Flavones	Luteolin, Diosmin, Apigenin, Wogonin	Broccoli, onions, oranges, parsley, grapefruit, cabbage, and carrot
Flavonols	Quercetin, Morin, Galangin, Kaempferol	Tea, apples, onions, broccoli, strawberries, leeks, and grapefruits
Anthocyanins	Malvidin, Cyanidin, Hirsutidin, Pelargonidin	Kidney beans, red wine, and berry fruits
Isoflavones	Genistein, Glycitein, Daidzein, Equol	Soy and soy products

**Table 2:** Promising preclinical studies of flavonoids and their neuroprotective role against Alzheimer’s disease.

Flavonoids	Models	Concentrations	Effects	References
Quercetin	3xTg-AD mice model	100 mg/kg	Reduces Aβ protein, tauopathy in hippocampus and amygdala	[106]
Naringin	Intracerebroventricular (ICV) streptozotocin (STZ) induced-cognitive impairment in rat	50, 100 and 200 mg/kg	Improves mitochondrial dysfunction-induced oxido-nitrosative stress as well as inflammatory surge	[107]
Naringenin	ICV STZ-induced dementia model of rats	25, 50 mg, 100 mg/kg	Reduces brain Aβ levels and reversed tau hyper-phosphorylation through downregulation of glycogen synthase kinase-3β (GSK-3β) activity in hippocampus and cerebral cortex	[108]
Nanoparticle of epigallocatechin-3-gallate	APPswe/PS1dE9 mice	-	Increase in synapses, and reduction in neuroinflammation as well as Aβ plaque burden	[109]
Epicatechin	PC12 cells treated with Aβ_25–35_	10 µM	Reduces Aβ-induced neurotoxicity	[110]
Catechin	PC12 cells treated with Aβ_25–35_	10 µM	Reduces Aβ-induced neurotoxicity	[110]
Luteolin	STZ-induced AD rat	10 and 20 mg/kg	Improves spatial learning and memory impairment	[111]
Diosmin	3xTg-AD mice	1 and 10 mg/kg/day	Enhances inhibitory GSK-3β phosphorylation and lessen γ-secretase activity, Aβ generation, as well as tau hyperphosphorylation	[112]
Wogonin	3xTg-AD mice	10 mg/kg	Attenuates amyloidogenic pathway and increased mitochondrial membrane potential and protected against apoptosis	[113]
